# Secondary distress in violence researchers: a randomised trial of the effectiveness of group debriefings

**DOI:** 10.1186/s12888-017-1327-x

**Published:** 2017-06-02

**Authors:** Heidi Grundlingh, Louise Knight, Dipak Naker, Karen Devries

**Affiliations:** 1 Social and Mathematical Epidemiology Group, Gender Violence and Health Center, Department of Global Health and Development, London School of Hygiene and Tropical Medicine, London, UK; 2 Raising Voices, Kampala, Uganda

**Keywords:** Secondary distress, Emotional distress, Vicarious trauma, Secondary traumatic stress, Violence, Debriefing, Trial, Epidemiology, Researchers

## Abstract

**Background:**

Secondary distress including emotional distress, vicarious trauma (VT) and secondary traumatic stress (STS) due to exposure to primary trauma victims have been described in helping professionals and in violence researchers. To our knowledge, there are few prevalence studies, and no tailored interventions have been tested to reduce secondary distress in violence researchers. The study aims to (1) describe the epidemiology of secondary distress experienced by violence researchers; to (2) assess the effectiveness of group debriefings in mitigating secondary distress; to (3) assess risk and protective factors.

**Methods:**

We conducted an un-blinded, individually randomised trial with parallel assignment. Eligible participants were 59 Ugandan researchers employed by the Good Schools Study to interview children who experienced violence in a district of Uganda. Fifty-three researchers agreed to participate and were randomly allocated. The intervention group (*n* = 26) participated in three group debriefings and the control group (*n* = 27) in three leisure sessions (film viewings). The primary outcome was change in levels of emotional distress (SRQ-20); secondary outcomes were levels of VT and STS at end-line. A paired t-test assessed the difference in mean baseline and end-line emotional distress. Un-paired t-tests compared the change in mean emotional distress (baseline vs. end-line), and compared levels of VT and STS at end-line. Separate logistic regression models tested the association between end-line emotional distress and a-priori risk or protective factors.

**Results:**

Baseline and end-line levels of emotional distress were similar in control (*p* = 0.47) and intervention (*p* = 0.59) groups. The superiority of group debriefing over leisure activities in lowering levels of emotional distress in the intervention group (*n* = 26; difference in SRQ-20 = 0.23 [SD = 2.18]) compared to the control group (*n* = 26; difference in SRQ-20 = 0.23 [SD = 1.63]) could not be detected (*p* = 1). In regression analysis (*n* = 48), baseline distress increased the odds of end-line distress (OR = 16.1, 95%CI 2.82 to 92.7, *p* = 0.002). Perceived organisational support (OR = 0.09, 95%CI 0.01 to 0.69, *p* = 0.02) and belief in God (OR = 0.21, 95%CI 0.03 to 1.26, *p* = 09) was protective against end-line distress.

**Conclusion:**

We found no evidence that violence researchers experienced elevated emotional distress after doing violence research. There was no difference between group debriefings and leisure activities in reducing distress in our sample. However, the hypotheses presented should not be ruled out in other violence research settings. Our findings suggest that organisational support is a significant protective factor and belief in God may be an important coping mechanism.

**Trial registration:**

Clinical Trials NCT02390778. Retrospectively registered 19 March 2015. The Good Schools Trial was registered at (NCT01678846), on August 24, 2012.

**Electronic supplementary material:**

The online version of this article (doi:10.1186/s12888-017-1327-x) contains supplementary material, which is available to authorized users.

## Background

Greater recognition of the health burden of disease caused by violence against women and children continues to provide impetus for better provision of care but also further research in the field [1–4]. As researchers engage empathetically with victims of violence and abuse, they may experience secondary distress which impacts their mental wellbeing and potentially compromises field research [5–8]. Increasingly, large scale research surveys include items on the experience of violence and in-depth violence research proliferates in many settings. However, there is little epidemiological research which investigates secondary distress in researchers who engage with victims of violence, or experimental research which tests group interventions to prevent potential deleterious effects in professionals. In line with previous research, we defined ‘violence researchers’ as researchers who engage in face-to-face interviews with victims of violence (study participants) to collect quantitative or qualitative data on their experience of violence [5].

### Secondary distress: emotional distress, vicarious trauma and secondary traumatic stress

Secondary distress is a broad term, and refers to distress experienced by those providing professional care to, or otherwise engage with, people who have been primary victims of a trauma. Emotional distress, vicarious trauma and secondary traumatic stress are related types of secondary distress and can have psychological, cognitive and physical impact on those who work with trauma victims [5, 9, 10]. Studies which measure a combination of these outcomes provide more comprehensive insight [9, 11].

Emotional distress has been described in social workers and nurses who counsel victims of trauma [12, 13]. Emotional distress is well documented in women who suffer domestic abuse [14, 15] and manifests as psychological symptoms of distress (feeling worried or tense, headaches, poor digestion, tiredness), including depression and anxiety [16]. Emotional distress may be the mild or early manifestation of poor mental health. Vicarious trauma was recently qualitatively described in sexual violence researchers [5] and in qualitative researchers who conduct interviews with children who have suffered abuse [7]. It is theorised that empathetic listening to a victim’s traumatic experiences or witnessing their suffering and pain may evoke ‘intense emotions such as profound sadness, helplessness, frustration, and anger’ [17]. This emotional response may in turn lead to vicarious trauma, characterised by negative changes in a therapist’s or helper’s cognitive schemas i.e. their view of themselves, others and the world [18]. In one study, violence researchers reported ‘a deep change in me, physically, psychologically and cognitively’ as awareness grew ‘that there are perpetrators who live, work and play among us who are capable of inflicting such atrocities…’ [5]. Secondary Traumatic Stress is described as a ‘natural consequence of caring between two people, one of whom has been initially traumatized and the other whom is affected by the first’s traumatic experiences’ [19]. Figley further defined secondary trauma as ‘a syndrome of symptoms parallel to those of post-traumatic stress disorder (PTSD)’ [20]. STS has been described in qualitative and quantitative studies of nurses [19], forensic interviewers and violence researchers [5, 21], social workers [22], attorneys [23] and mental health professionals [24]. Researchers may experience nightmares, sleep disturbances and palpitations (hyper arousal), avoiding situations that remind them of the trauma narratives (avoidance) or may re-experience trauma victims’ thoughts or feelings (intrusive symptoms) [5, 9].

Although there is increasing recognition for the risk of secondary distress in helping professionals, there are few systematic and empirical studies of secondary distress, with some studies indicating a moderate association with professionals’ exposure to stories of trauma and secondary distress, i.e. manifesting as emotional distress, vicarious trauma or secondary traumatic stress [9, 25, 26]. Secondary distress in violence researchers has been described through early qualitative work, but the epidemiology, including how many researchers may be at risk, is yet to be investigated. Although we have a growing understanding of the risk and protective factors, further work is needed to understand the interplay and associations, for example between personal characteristics, organizational support or coping strategies [27, 28]. To our knowledge, no experimental studies investigate the effectiveness of targeted interventions to prevent secondary distress in violence researchers.

### Group debriefing in mitigating the effect of trauma and distress

In recent systematic reviews, the randomised control trials which assess the efficacy of single session group psychological debriefing show varied results, with early reviews suggesting no benefit [29, 30]. Reviewers note that the methodological quality of primary studies was poor and suggest an ‘urgent need for randomised controlled trials of group debriefing and other early interventions’ [29]. Critical Incident Stress Debriefing (CISD) is one such group debriefing method.

CISD supports the ‘normal recovery process and adaptive functions in psychologically healthy professionals’ and provides a structured group story-telling process and practical information to normalise group member reactions to a critical incident [31, 32]. It has been widely used for the prevention of post-traumatic stress disorder in first responders after a single traumatic event [31, 33] and has been shown to be effective with nursing, emergency and military personnel [34–36]. The potential benefit of CISD-type group debriefings in mitigating secondary distress in violence researchers is yet to be empirically assessed.

The proponents of CISD suggest the method differentiates itself from other group psychological debriefing methods, in that it should be used within a supportive organisational setting, with psychologically healthy professionals and provided by an experienced facilitator following the methodological guidelines outlined for CISD. Based on the dearth of tested interventions possibly suited to a low resource setting, we looked to the CISD approach to develop a three session intervention (‘Group Debriefing for Secondary Distress’) with consideration for the parameters suggested by CISD proponents [37].

Cognisant of these knowledge gaps, the current study was conducted with violence researchers employed by the Good Schools Study (end-line) to interview 5362 participants on their experiences of interpersonal violence [38]. Results from the Good Schools Study (end-line) show that between 60.2% (intervention schools) to 80.5% (control schools) of students interviewed by violence researchers reported past term physical violence at end-line [39]. Levels of emotional and sexual violence are yet to be published for the end-line Good School Study. Previously, more than 48.6% of students reported lifetime emotional violence while 11.8% of girls and 2.5% of boys reported life time sexual violence during the baseline Good Schools Study [40].

The activities of the Good Schools Study provided the opportunity to observe the epidemiology of secondary distress (including emotional distress, vicarious trauma and secondary traumatic stress) in a sizeable sample of violence researchers and conduct an exploratory trial to test the effectiveness of group debriefings. The findings from testing the following hypotheses are reported: (Hypothesis 1) Ugandan violence researchers experience increased emotional distress during a 5 week period of intensive interviewing of children and adults who disclose experiences of physical, emotional or sexual violence; *(*Hypothesis *2)* Ugandan violence researchers attending group debriefing sessions have lower levels of emotional distress, vicarious trauma and secondary traumatic stress as compared to their control group; (Hypothesis 3) Risk factors (baseline emotional distress, personal trauma history, number of referred and perceived primary trauma cases interviewed) are associated with higher levels of end-line emotional distress and protective factors (years paid work experience, organisational support, coping mechanisms) are associated with lower levels of end-line emotional distress in Ugandan violence researchers.

## Methods

### Trial design and procedure

The study was an individually randomised trial with parallel assignment. Eligible participants were 59 Ugandan research assistants employed by the Good Schools Study (GSS). Research assistants engaged with research participants as violence researchers. Over the course of 5 weeks, violence researchers conducted face to face interviews with 3943 children (potential victims of violence), as well as 591 teachers/school staff and 828 parents (potential perpetrators or victims of violence) on the experience of physical violence (including corporal punishment), emotional and sexual violence by teachers, students, family and others. Survey items included behaviourally specific acts of violence, for example, have you ever been insulted, cursed, hit, slapped, punched, choked, burnt, forced to do sexual things and so on. The research assistants were mostly university qualified professionals in their late twenties; some had previous experience in administering violence-related surveys.

Recruitment into the current trauma study was on the 15th and 16th of June 2014, via invitation to all researchers during formal information sessions. A total of 53 researchers/data capturers agreed to participate and all were enrolled into the study once written voluntary consent was obtained, providing the study with a near complete sample. After randomisation, the baseline survey was conducted before the researchers started interviewing children about their experiences of violence and the end-line survey was conducted after 5 weeks of interviewing children. As all violence researchers had a high level of proficiency in English the questionnaires were administered in English and self-completed by the violence researchers. The baseline assessment was on the 17th of June 2014 and end-line assessment on the 11th of July 2014. The complete study protocol is available as Additional file 1. We made two amendments to the protocol. All six data entrants for the study were originally invited to participate. Five declined, and the one who was accepted was randomised and completed data collection. This participant was subsequently excluded from analysis, because they did not collect data on violence directly and did not enter any violence related data. We also added two additional secondary outcome measures before the commencement of the trial to the end-line survey to assess levels of secondary traumatic stress. All information regarding the randomised trial are reported as per the CONSORT 2010 checklist (see Additional file﻿ 2).

### Ethics statement

Ethical approval for the current study (#8118) and the GSS trial (#6183) was obtained from London School of Hygiene and Tropical Medicine Ethics Committee and from the Uganda National Council for Science and Technology (SS 2520). The ethics committee approved a written informed consent procedure for the current study participants; no children or minors were enrolled in the current study. The study was registered as a clinical trial after completion, due to time constraints. The authors confirm that all on-going and related trials for intervention are registered. This trial is registered at www.clinicaltrials.gov (NCT02390778). The Good Schools Trial is registered at www.clinicaltrials.gov (NCT01678846).

Written informed consent to participate in the study and to publish the data was obtained. The baseline and end-line surveys were administered in June and July 2014 respectively. The surveys were administered at the field station of the Good Schools Study, which was located in the district of Luwero (Uganda). The questionnaire was programmed into mobile phones and was self-completed by the researchers, enabling them to do so in private locations e.g. their hotel room. All data was fully anonymised before analysis to prevent identification of individuals.

### Randomisation

Simple equal randomisation was used with a 1:1 allocation ratio to assign participants to either the intervention or control group. All participating violence researchers were invited to place a piece of paper with their name into an opaque paper bag. A volunteer from the group blindly selected one paper at a time from the container and these names were then allocated one-by-one either to the intervention (*n* = 26) or control group (*n* = 27). Due to the nature of the intervention, participants should be considered unblinded to group allocation.

### Intervention

The intervention, Group Debriefings for Secondary Distress, was designed for this study based on available current literature and tailored to violence researcher needs. A portion of content for the sessions was drawn from the “Critical Incident Stress Debriefing” (CISD) technique [31, 32]. The intervention involved story-telling, identifying emotional responses to these stories, psycho-education and practical information to normalise group member reactions to a distressing event. The intervention group participated in 3 consecutive face-to-face group debriefing sessions lasting 90–120 min each. Each session started with a fun ice-breaker to create a relaxed atmosphere and group cohesion. Session 1 focused on encouraging group participation, discussing primary trauma encountered and emotional reactions to these stories. Session 2 connected current experiences with the group members’ own trauma histories and life experiences. The last session focussed on societal and community responses to violence, and employing personal agency to find constructive ways to address violence in communities. Individuals were not pressured to disclose their experiences and could contribute anonymously through written postcards if preferred. Sessions were held in the hotel hall and scheduled after the work day, hence attendance was voluntary. The debriefings were designed and administered by HG, a health care professional with training and experience in facilitating health promotion activities in small groups. During the same time slot the control group was assigned to a leisure activity (film showing), for every session of debriefing undergone by the intervention group. The films were chosen for their light-hearted uplifting content and presented as a fun and relaxing activity.

All violence researchers were given the contact information for independent support services (for example non-profit organisations offering counselling or domestic violence support) that they could contact at any time without the GSS team knowing [41]. An outline of the group debriefing sessions is provided in the Additional file 3.

### Trial outcomes

We measured secondary distress via the three related types of distress described in the literature i.e. emotional distress, vicarious trauma and secondary traumatic stress. The primary outcome for emotional distress was the change in mean levels of emotional distress i.e. baseline vs. end-line, as measured by the Self-Report Questionnaire-20 (SRQ-20, 16]. The secondary outcome for vicarious trauma was mean levels of vicarious trauma at end-line, as measured by the Vicarious Trauma Scale (VTS) [42]. The secondary outcome for secondary traumatic stress was mean levels of secondary traumatic stress at end-line, as measured by the Impact of Event Scale-Revised (IES-R) [43] and the Professional Quality of Life Scale (ProQOL) Secondary Traumatic Stress subscale [44].

### Survey measures

The baseline survey included various categorical questions related to participant demographics, and a-priori risk and protective factors e.g. work experience and lifetime experiences of violence. Experiences of violence were measured using items from the WHO Multi Country Study on Women’s Health and Domestic Violence and included exposure to physical, sexual, and emotional violence from partners and non-partners [45]. The latter showed validity and strong internal consistency in Brazilian populations [46]. Items from measure were translated and carefully pretested for an earlier trial among Ugandan populations [47]. The Self-Report Questionnaire-20(SRQ-20) was included to assess baseline emotional distress. It has been widely used and validated among East African populations as a measure of mental health and wellbeing [16, 45, 48, 49].

The end-line survey included categorical questions related to the number and types of participant trauma cases perceived as distressing. We probed for ‘perceived primary trauma cases’ by asking questions such as: “During the past 5 weeks, which kind of reports from children did you find MOST upsetting or distressing?” End-line categorical questions further included a-priori risk and protective factors e.g. organisational support and coping mechanisms. Researchers were asked whether they experienced various forms of organisational support from the GSS management and a composite variable for this was generated. Agreement with 3 out of 5 statements was scored as high levels of perceived organisational support. The SRQ-20, Vicarious Trauma Scale (VTS), Impact of Events Scale-Revised (IES-R) and Professional Quality of Life Scale (ProQOL) were included to assess end-line secondary distress. The Vicarious Trauma Scale (VTS) has recently been developed for use as a screening tool for vicarious trauma in low resource settings and had high internal reliability during testing, but validity has not been established [11, 42]. It is the only publicly available scale for vicarious trauma and hence selected as a measure. The Impact of Events Scale-Revised (IES-R) scale yielded high reliability and acceptable validity in several populations and is widely used to screen for post-traumatic stress disorder (PTSD) and recently to test for secondary traumatic stress [43, 50, 51]. The Professional Quality of Life Scale (ProQOL) was developed to screen mental health and other professionals who may experience positive or negative impacts as they help others. Versions of the screening tool yielded good construct validity and high reliability in various countries and it is widely used in research as a measure for secondary traumatic stress and related constructs [19, 44]. The cut points for the SRQ-20 is ≥6, the IES-R is ≥33 and the ProQOL is ≥56; the VTS cut point has not been established [43, 44, 49]. The cut points for these measures indicate the score at which further referral and assessment is recommended.

The average number of interviews conducted per interviewer was ascertained from the Good School Schools (GSS) trial data. During the GSS trial, real time algorithms were applied to the incoming GSS trial data to identify primary trauma cases in need of referral. The criteria related to the severity of recent acts of violence and/or visible and significant emotional distress experienced by study participants. As these referral cases could cause secondary distress in researchers they were included for analysis as ‘referred primary trauma cases’.

Most of the variables related to the baseline or end-line characteristics of participants were dichotomised based on yes/no responses. For the number of ‘referred’ or ‘perceived’ trauma cases seen, we used equal frequency grouping intervals to create categorical variables for comparison. The SRQ-20, VTS, IES-R and ProQOL scores were modelled as continuous and mean group scores obtained for analysis. Consistent with previous research the SRQ-20 scores were also dichotomised with the top 33% of the sample deemed as having a ‘high’ score indicative of probable depression/anxiety [1].

### Data analysis methods

All analyses were conducted by using STATA/IC 13.0. The descriptive end-line characteristics were compared using the chi-square and the Fishers exact test to compare proportions and the t-test to compare continuous variables. To test hypothesis 1, a paired t-test was performed to assess if there is a significant difference in the mean SRQ-20 scores between baseline and end-line, within each of the control and intervention groups. The study had 90% power to detect a 1.25 point difference in base and end-line SRQ20 scores. To test hypothesis 2, a mean unpaired t-test was performed to compare the change in mean SRQ-20 score (baseline vs. end-line) for the controls with the change in mean SRQ-20 score for the debrief group. The analysis further included unpaired t-tests to compare mean levels of vicarious trauma and secondary traumatic stress between the two groups at end-line as measured by the VTS, IES-R and ProQOL scales.

We compared mean outcome levels of distress in control and interventions groups, rather than dichotomising participants in the groups around the median or cut points established for the various measures. Firstly, statistical power to detect a difference between groups is reduced when dichotomising a continuous variable [52]. In addition, dichotomizing around a cut point would limit our analysis to comparing those individuals who needed referral with those who did not. (The cut points for our measures indicate the score at which further referral and assessment is recommended.) Analysing mean outcomes provided a more nuanced understanding of the effect of exposure and intervention within the groups. For example, participants may experience a significant increase in their level of distress over time due to exposure and although it may be below a level requiring referral at individual level, it would still indicate the need for preventative measures or professional support at group level. A mean difference in secondary distress due to an intervention would also indicate promising results, warranting further research.

The primary analysis was intention to treat and included 52 participants (intervention group *n* = 26, control group *n* = 26) who were randomly assigned. The single data entrant was excluded from the control group in analysis, because they did not collect data on violence via face to face engagement. For those lost to follow up, the baseline SRQ-20 score was carried forward as an end-line score under the conservative assumption that these participants would have the same individual SRQ-20 score at end-line as they had at baseline. The VTS, IES-R and ProQOL measures are designed for end-line (post exposure) assessment and hence the mean end-line VTS, IES-R and ProQOL scores of each group was imputed for those participants who were lost to follow-up. This was under the assumption that they would have similar mean scores as seen in their respective groups at end-line.

A per protocol analysis (*N* = 48, intervention group *n* = 22, control group *n* = 26) was conducted for all participants which attended at least 2 out of the 3 sessions of group debriefings or leisure sessions.

To investigate hypothesis 3, separate logistic regression models were fitted to test the association between experiences of end-line emotional distress in Ugandan researchers and selected a-priori risk or protective factors. Emotional distress (SRQ-20 score) was modelled as a binary variable with top 33% of sample deemed as having a ‘high’ score. Confounding covariates included in the adjusted model were selected a priori and included age, sex, baseline emotional distress and participation in control or debrief group. Age had an insignificant effect in modelling and was therefore not retained in some models to allow for greater power. This analysis included all researchers who completed the trial apart from the single data entrant who was excluded (*n* = 48).

## Results

### Descriptive findings

**Fig. 1:**
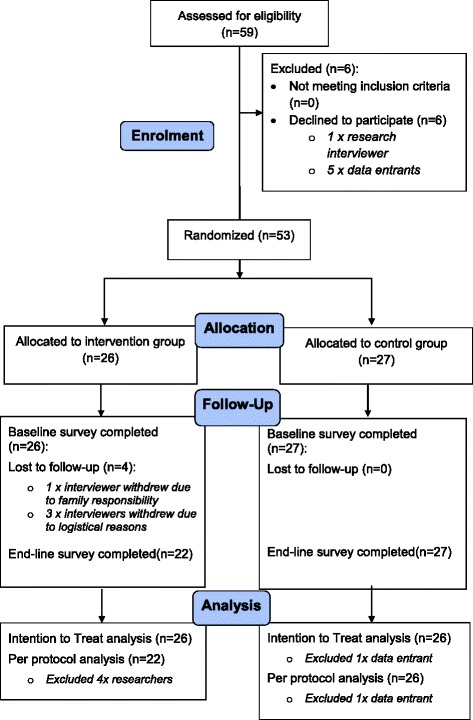
Participant flow diagram

Of the 59 eligible participants, 53 agreed to participate and were randomly allocated to either receive group debriefing (intervention group, *n* = 26) or participate in a leisure activity (control group, *n* = 27). Four of the enrolled violence researchers allocated to the intervention group were lost to follow-up due to reasons unrelated to the intervention. Three violence researchers could not participate in the debriefing sessions due to logistical reasons and then withdrew from the study. One researcher resigned from the GSS due to family responsibilities. 49 researchers completed the trial; *n* = 27 in the control group and *n* = 22 in intervention group. Only one of the group debriefing participants could not attend one out of three sessions due to illness. All the control group participants attended the leisure sessions. One data entrant was excluded from analysis and hence the control (*n* = 26) and interventions group (*n* = 26) analysis was conducted accordingly. See Fig. 1 for the participant flow diagram.

The baseline summary statistics are presented by control and intervention group in Table 1. The groups had similar proportions of female (and male) participants. Both groups were constituted of younger professionals with the average age per groups estimated at 29.5 and 30 years respectively. 88% or more of the researchers had university degrees, while intervention group members reported more years paid work experience. The groups had similar proportions of participants reporting lifetime experience of violence and high baseline emotional distress.

**Table 1:** Baseline and end-line demographic characteristics

Baseline demographics characteristics^a^	Control (*n* = 26)		Intervention (*n* = 26)		*P* value
	No.	%	No.	%	
Demographics					
Gender (Female)	19	73%	15	58%	0.24
Age, mean years (SD)	29.5	(4.49)	30	(4.47)	0.69
Highest qualification					
University Degree	25	96%	23	88%	-
Certificate or Diploma	1	4%	3	12%	0.61
Paid work experience (>5 years)	9	35%	16	62%	0.05
Personal Experience of Violence (lifetime)					
Intimate partner violence(emotional, sexual or physical)	7	27%	5	19%	0.51
Sexual violence from others	2	8%	1	4%	0.50
Baseline emotional distress (SRQ-20)					
Violence researchers scoring at top 33% of the sample	5	19%	7	32%	0.31
End-line characteristics^b^	Control (*n* = 26)		Intervention (*n* = 22)		*P* value
	No.	%	No.	%	
Levels of exposure to secondary trauma (5 week trial)					
Mean no. of interviews(child) per interviewer (SD)	74	(48.1)	86	(45.2)	0.66
No. of 'referred primary trauma cases' (child)					
0 to 3 cases	9	35%	5	23%	
4 to 13 cases	9	35%	8	36%	
14 to 41 cases	8	31%	9	41%	0.63
No. of 'perceived primary trauma cases'(child/adult)					
none	4	15%	1	5%	
1 or 2 cases	11	42%	5	23%	
3 to 20 cases	11	42%	16	73%	0.13
Cases found MOST distressing (choice of two options^c^)					
sexual violence	17	65%	11	50%	0.28
imminent sexual violence	1	4%	5	23%	0.08
physical violence	12	46%	15	68%	0.13
imminent physical violence	0	0%	1	5%	0.45
emotional violence/bullying	5	19%	9	41%	0.10
corporal punishment	8	31%	11	50%	0.18
material need	7	27%	5	23%	0.74
domestic violence	4	15%	4	18%	1.00
Perceived levels of organisational support (5 week trial)					
Composite perceived levels of organisational support^d^	21	81%	19	86%	0.60
We had regular staff meetings.	11	42%	9	41%	0.92
There was colleague at work I could talk to.	21	81%	18	82%	0.48
While working for GGS, I felt I was part of a team.	23	88%	21	95%	0.38
I felt that my employer cared about my wellbeing.	19	73%	14	64%	0.48
I could talk to my supervisor when unhappy at work.	20	77%	16	73%	0.73
Coping Strategies (5 week trial)					
In order to cope with my job as a researcher I have used:					
Support of family and friends	14	54%	6	27%	0.06
Support of colleagues	21	81%	20	91%	0.32
Support of supervisor	16	62%	17	77%	0.24
Exercise or physical activity	5	19%	3	14%	0.71
Personal belief in God	18	69%	18	82%	0.32
Spending time alone/relaxing activity	14	54%	8	36%	0.23
Music	13	50%	11	50%	1.00
Watching television	14	54%	6	27%	0.06
Medication to alleviate symptoms of stress	8	31%	8	36%	0.68
End-line emotional distress (SRQ-20)					
Violence researchers scoring at top 33% of the sample	8	31%	6	27%	0.79

SD standard deviation indicated for mean value

^a^Per protocol analysis of end-line characteristics (*n* = 48)

^b^Intention to treat analysis of baseline characteristics (*n* = 48)

^c^interviewers asked to indicate the two most distressing kind of reports

^d^agree with 3/5 statements

Table 1 also provides detailed end-line summary statistics. Over the course of 5 weeks, the GSS researchers interviewed 3931 children and interviews lasted between 90 to 60 min. Levels of exposure to research participants and therefore possible secondary trauma due to participants’ reports of violence are similar for both groups. During the same period both groups had similar levels of ‘referred primary trauma cases’. When asked how many cases they ‘found upsetting or distressing’, a higher number of intervention group members (73%) reported interviewing between 3 to 20 ‘perceived primary trauma cases’ compared to controls (42%), although this did not reach significant levels (*p* = 0.13). Both groups found cases of sexual or physical violence or corporal punishment particularly distressing, while a higher number (approaching significance, *P* = 0.8) of intervention members reported cases where ‘sexual violence may be imminent’ as distressing. Both groups reported high levels of organisational support from colleagues and supervisors. Controls report receiving more support from their family (approaching significance with *P* = 0.06). Watching TV was an important coping mechanism for controls, which may relate to the intervention they received (approaching significance with *P* = 0.06). The groups had similar proportions of members reporting higher levels of end-line emotional distress.

### Levels of emotional distress in Ugandan researchers

Levels of emotional distress over time were investigated in the control and intervention groups to test Hypothesis 1. Table 2 shows the estimated difference in SRQ-20 scores over time were not significant in either the control (difference in SRQ-20 = 0.23 [SD = 1.63], *p* = 0.47) or the debrief group (difference in SRQ-20 = 0.23 [SD = 2.18], *p* = 0.59), indicating that levels of emotional distress remained unchanged over the course of the study.

**Table 2:** Change in emotional distress

Paired t test	*n*	Mean	StDev	SE mean	CI (95%)	*P* value
Control group (*N* = 26), ITT						
SRQ20 score at baseline	26	2.38	1.89	0.37	(1.61; 3.15)	-
SRQ20 score at end-line	26	2.15	1.80	0.35	(1.42; 2.88)	-
Difference in SRQ20 score	26	0.23	1.63	0.32	(−0.42; 0.89)	0.47
Debrief group (*N* = 26), ITT						
SRQ20 score at baseline	26	2.57	1.7	0.33	(1.88; 3.26)	-
SRQ20 score at end-line	26	2.34	2.36	0.46	(1.38; 3.30)	-
Difference in SRQ20 score	26	0.23	2.18	0.43	(−0.64; 1.11)	0.59

*ITT* intention to treat analysis

### The effectiveness of group debriefings

Levels of emotional distress, vicarious trauma and secondary traumatic stress were compared in intervention and control groups to test Hypothesis 2. The intention to treat analysis (ITT) showed no statistical difference in ‘change in SRQ-20 score’ when comparing the control (difference in SRQ-20 = 0.23 [SD = 1.63]) and intervention groups (difference in SRQ-20 = 0.23 [SD = 2.17]), *p* = 1 (see Table 3). The intervention group showed a slightly higher but non-significant VTS score (ITT analysis: *p* = 0.41) and significantly higher IES-R score (ITT analysis: *p* = 0.03) and ProQOL Secondary Traumatic Stress subscale score (ITT analysis: *p* = 0.02) versus the control group.

**Table 3:** Comparing secondary distress in control and intervention groups

Secondary distress outcomes	Group								
	Control			Intervention			Mean difference		
	n	Mean	SD	n	Mean	SD	Mdif	95% CI	*P*
Emotional distress									
SRQ20, total score (baseline)^a^	26	2.38	1.89	26	2.57	1.7			
SRQ20, total score (end-line)^a^	26	2.15	1.80	26	2.34	2.36			
SRQ20, change in score (basel vs. end)^a^	26	0.23	1.63	26	0.23	2.17	0	−1.07;1.07	1
SRQ20, total score (baseline)^b^	26	2.38	1.89	22	2.64	1.81			
SRQ20, total score (end-line)^b^	26	2.15	1.80	22	2.36	2.56			
SRQ20, change in score (basel vs. end)^b^	26	0.23	1.63	22	0.27	2.37	0.04	−1.21;1.13	0.94
Vicarious trauma									
Vicarious Trauma Scale (VTS) total score^a^	26	19.9	4.89	26	21	4.04	−1.03	−3.53; 1.46	0.41
Vicarious Trauma Scale (VTS) total score^b^	26	19.9	4.89	22	21	4.41	−1.03	−3.75; 1.69	0.45
Secondary trauma									
Impact of Events Scale-R (IES-R) total score^a^	26	14.03	9.67	26	19.6	8.24	−5.55	−10.5; −.54	0.03
Impact of Events Scale-R (IES-R) total score^b^	26	14.03	9.67	22	19.6	8.9	−5.55	−11.5; −0.59	0.05
Secondary trauma (ProQOL subscale)									
Secondary Traumatic Stress Subscale^a^	26	50.23	7.13	26	54.4	4.58	−3.98	−7.32;-0.64	0.02
Secondary Traumatic Stress Subscale^b^	26	50.23	7.13	22	54.4	4.99	−3.95	−7.59;-0.31	0.03

^a^Intention to treat analysis

^b^Per protocol analysis

### Risk or protective factors associated with emotional distress

The association between emotional distress and a priori factors were tested as per Hypothesis 3. Table 4 shows the odds of high levels of end-line emotional distress in Ugandan researchers who have been exposed to various factors, relative to the odds of high levels of end-line emotional distress in those who have not been exposed to these factors. Models unadjusted and adjusted for potentially confounding characteristics are presented. After adjusting for baseline emotional distress, sex and group allocation, researchers were less likely to report end-line emotional distress when they perceived organisational support (OR = 0.09, 95%CI 0.01 to 0.69, *p* = 0.02) from the Good School Study management/supervisory staff and reported belief in God (OR = 0.21, 95%CI 0.03 to 1.26, *p* = 09) as a coping mechanism. Researchers with elevated levels of baseline distress are significantly more likely to have elevated levels of end-line emotional distress (OR = 16.1, 95%CI 2.82 to 92.7, *p* = 0.002). Those with end-line emotional distress were more likely to use medication to alleviate symptoms of stress (OR = 18.9, 95%CI 2.76 to129.27, *p* = 0.003). Exposure to referred or perceived trauma cases and other variables were not associated with high levels of end-line emotional distress.

**Table 4:** Association between end-line emotional distress and various factors

Elevated end-line emotional distress	Unadjusted OR (*N* = 48)			Adjusted OR (*N* = 48)		
	UOR	95% CI	P	AOR	95% CI	*P*
Elevated baseline emotional distress^a^	10	2.26;44.02	0.002	16.1	2.82;92.7	0.002
Personal trauma history, lifetime						
Intimate partner violence or non-partner sexual violence^b^	2.43	0.64;9.14	0.188	0.86	0.15;4.76	0.86
Paid work experience (≥ 5 years)^b^	0.95	0.27;3.33	0.94	1.14	0.26; 5.03	0.85
Referred primary trauma cases, past 5 week^b^						
0 to 7 cases	1			1		
8 to 41 cases	1.01	0.27;3.73	0.97	0.67	0.14, 3.24	0.62
Perceived primary trauma cases, past 5 weeks^b^						
0 to 2 cases	1			1		
3 to 20 cases	0.46	0.13;1.64	0.23	0.89	0.19;4.11	0.88
Perceived organisational support^c^, past 5 weeks^b^	0.17	0.03;0.87	0.03	0.09	0.01;0.69	0.02
Coping mechanisms^b^						
Support of family and friends	1.07	0.30;3.77	0.91	2.6	0.43;15.62	0.29
Personal belief in God	0.28	0.07; 1.13	0.07	0.21	0.03; 1.26	0.09
Spending time alone/music/television	2.9	0.79; 10.6	0.1	2.57	0.55; 11.9	0.22
Medication to alleviate symptoms of stress	11.6	2.71; 50.07	0.001	18.9	2.76; 129.27	0.003

^a^Modelled separately, adjusted for age, sex and participation in control or debrief group.

^b^Modelled separately, adjusted for sex, participation in control or debrief group and baseline emotional distress.

^c^Composite perceived levels of organisational support (agree with 3/5 statements)

## Discussion

We found no evidence that Ugandan violence researchers administering a violence survey experienced elevated emotional distress and no evidence that a group debriefing intervention was any more effective in reducing secondary distress than group leisure activities. However, we found two a priori factors were associated with decreased end-line distress in individuals: perceived organizational support and belief in God.

Contrary to Hypothesis 1, Ugandan violence researchers’ average level of emotional distress as measured by the SRQ-20 remained stable in both our control and intervention groups. During an intensive 5-week period of violence research, the baseline and end-line scores ranged between 2.15 and 2.64 for both groups and produced a non-significant difference. The validated SRQ-20 cut points for the Ugandan population and for sub-Saharan countries are ≥6 and ≥7 out of 20, respectively, with scores exceeding these indicative of possible emotional distress, requiring further referral and assessment [48, 49]. Hence, compared to other samples, the levels of emotional distress in this sample may have been low.

Our sample may have maintained low levels of emotional distress due to the relatively short period (5 weeks) of exposure to reports of violence. The mean baseline SRQ-20 scores in our sample of violence researchers were quite low, indicative of relatively good mental health. In a group with good baseline mental health, it may take some time and cumulative exposure before significant emotional distress (characterised by physiological symptoms) manifest, as also suggested by qualitative findings [5, 13, 53].

Violence researchers may have perceived the violence disclosed to them as ‘normal’, further maintaining low levels of emotional distress. In the study context, over 90% of Ugandan primary school students report lifetime experience of physical violence from teachers and more than 50% report past week experiences [40]. Despite this, the violence researchers perceived only some of the interviews with study participants as distressing (Table 1). Cultural norms around the acceptable use of corporal punishment with children may have normalised the experience for these researchers and prevented significant emotional distress.

In addition, it is likely that the type of violence reported to violence researchers matters. Previous qualitative research indicates that violence researchers found qualitative interviews with victims of sexual abuse particularly distressing [5, 10]. In our study the researchers also perceived severe sexual violence as especially distressing, however very few cases of severe sexual violence were reported to the researchers [40]. Limited exposure to reports of sexual violence may prevent secondary distress. Although emotional distress was not detected in this sample of researchers interviewing children attending primary school, violence researchers also engage with other populations such as child soldiers, former abductees or war victims. These different types of victims and settings may cause secondary distress and warrants further testing.

Hypothesis 2 proposed that violence researchers attending group debriefing sessions would have lower levels of emotional distress, vicarious trauma and secondary traumatic stress. In our sample the intervention group did not have lower levels of emotional distress, possibly because the levels of emotional distress in the control and debrief group did not change over time and was not elevated enough for the intervention to have a significant effect.

Contrary to Hypotheses 2, slightly higher but non-significant levels of vicarious trauma and significantly higher levels of secondary traumatic stress were observed in the intervention group at end-line. The relatively lower levels of vicarious trauma and secondary traumatic stress in the control group may have been affected by response bias such a lack of self-awareness, minimization or denial [54]. The relatively higher levels of vicarious trauma and secondary traumatic stress scores in the intervention group may maybe suggestive of heightened awareness in these participants due to the debrief sessions. The debrief group sessions asked intervention group participants to think and discuss the cases that ‘stayed’ with them and the effect violence may have on the long term development of children. Further discussions on coping mechanisms and the ability to remain resilient despite work pressures were facilitated. This may have heightened the awareness of intervention group members to the impact of violence on children and their own emotional response to such. Considering that the various scales include questions on recurring thought or feelings, our intervention may have raised the level of awareness in the intervention group members, elevating their response rates on the VTS, IES-R and ProQOL scales. Concurrently, heightened awareness may be on the pathway to fostering empathy, integrating experiences and improving adaptive functions. Further research is needed to explore these pathways in more detail.

Reviews comparing the efficacy of group debriefing on psychological distress identify a small number of studies which reported increased risk of psychological symptoms of distress [29, 55]. In the light of previous studies and our exploratory trial, future trials testing group debriefings should consider the potential risk to participants and mitigate these by noting the suggested parameters for use. These include facilitation by an experienced facilitator, following the proven methodological guidelines and using group debriefing in the context of organisational support rather than as a stand-alone activity [37].

Hypothesis 3 suggested associations between end-line emotional distress and a priori risk and protective factors. Despite the overall low levels of emotional distress observed in our group of violence researchers, we found significant relationships between some risk and protective factors and levels of emotional distress in individuals. The strong association between high levels of baseline and end-line emotional distress (*P* = 0.002) may indicate that researchers’ preceding mental health state was maintained throughout the study. Personal trauma history was not associated with high end-line emotional distress in this exploratory study, but should be investigated in future studies which observe more intense exposure of researchers to reports of sexual and severe physical violence [6, 10]. Previous findings support the notion that professionals with more years of professional working experience are more resilient to secondary distress [56]. In our sample, however, individual violence researchers with five or more years of paid work experience were not less likely to experience emotional distress.

We found no association between exposure to trauma cases (cases either referred due to experience of severe violence or perceived by researchers as distressing) and researcher levels of emotional distress. Factors such as type of violence and period of exposure, discussed under Hypothesis 1, may be at play.

Perceived organisational support was strongly associated with those researchers who reported lower levels of end-line emotional distress (*P* = 0.02). The Good School Study undertook extensive preparation for the violence study, including intensive training of violence researchers and field management. Researchers were grouped in small teams, each with a supportive supervisor. Regular supervisory and team meetings were held so all could voice their concerns and find mutual solutions. Organisational support (training, supervision, self-care, education, group support, work environment and general support from colleagues) as a protective factor is described in previous findings [53, 56, 57] and should be explored further as a means of preventing secondary distress in violence researchers.

Belief in God emerged as a near significant protective factor (*P* = 0.09), hence creating freedom to pursue faith-related practices may be beneficial in Ugandan populations. The positive associations with support from family and friends, leisure activities and taking medication (*P* = 0.003) may indicate that those with higher levels of distress were more likely to draw on social networks, leisure activities and medication as indicated by previous studies [5, 58, 59].

### Strengths and limitations

This is the first quantitative study to observe secondary distress in Ugandan violence researchers and may be the first to trial the superiority of group debriefings in mitigating secondary distress in researchers. This study highlights the importance of risk and protective factors associated with secondary distress. The Good Schools Study employed a large team of violence researchers and the sample size provided the study with 90% power to detect a 1.25 point difference in base and end-line SRQ-20 scores. In the light of the low overall levels of emotional distress detected in the groups over time, this may have provided enough power to detect a difference which is clinically significant and hence the conclusion that a meaningful difference in emotional distress would have been detected.

A limitation is that a single, standardised multi-item measure has not been developed to capture all the related concepts of secondary distress and, as with other studies, a combination of measuring tools was used. Although the IES-R and ProQOL have been validated in similar low income countries, they have not been validated in Ugandan populations and may therefore not be culturally appropriate.

The intervention group was instructed not to share the content of the sessions with the controls, but contamination cannot be ruled out as both groups shared close quarters at the hotel and during the work day. Further, the relaxation activity may have normalized distressing experiences in the controls through social engagement and providing escape. Reporting bias may have arisen in both groups; in the control group due to a combination of minimization, a lack of self-awareness and denial and in the intervention group due to their participation in group debriefings creating a heightened sense of awareness. This made comparison between the groups for true levels of perceived primary trauma cases and levels of secondary distress problematic.

## Conclusions

We found no evidence of elevated levels of secondary distress in violence researchers as a result of violence research. Group debriefings did not prove superior in mitigating levels of distress; however, both the control and interventions groups did not experience elevated levels of secondary distress. The sample of violence researchers was recruited for their ability to speak the local language, sensitivity towards participant experiences of violence and interviewing competence; hence, they are representative of short term researchers employed for quantitative interviews on violence in developing countries. Although this study is exploratory, findings are likely representative for similar settings and implies that it should not automatically be assumed that all violence researchers will experience secondary trauma during the course of their work.

This is one of the first quantitative studies to highlight that perceived organizational support and belief in God were strongly associated with lower end-line distress. This suggests that interventions which concentrate on providing tailored organisational support and personal time for religious activities may find this to be beneficial for preventing secondary distress in violence researchers.

Further work is needed to determine which individual researchers are at risk, the level and type of exposure to trauma victims that increases risk of secondary distress, what types of support are most appropriate and how and when this should be provided. It would also be beneficial to further understand the pathways by which organisational support, belief in God and other factors may relate to reduced risk of emotional distress in this population, so that effective support interventions can be developed. Finally, future trials in larger samples are needed to test the effect of interventions such as group debriefing and the process of building professional resilience.

## Additional files

Additional file 1: Study Protocol. (DOCX 28 kb)

Additional file 2: CONSORT 2010 Checklist. (DOC 217 kb)

Additional file 3: Outline: group debriefing sessions for secondary distress. (DOCX 13 kb)

## Abbreviations

GSS: Good Schools Study
IES-R: Impact of Events Scale – Revised
ITT: Intention to Treat Analysis
ProQOL: Professional Quality of Life Scale
PTSD: Post-traumatic Stress Disorder
SRQ-20: Self-Report Questionnaire-20
STS: Secovindary traumatic stress
VT: Vicarious trauma
VTS: Vicarious Trauma Scale
