# Supplementary material for: Secondary distress in violence researchers: a randomised trial of the effectiveness of group debriefings
Source: BMC Psychiatry. 2017 Jun 2;17:204. doi: 10.1186/s12888-017-1327-x (PMC5455179; doi:10.1186/s12888-017-1327-x)
Supplement: Supplementary file 1 — Study Protocol. (DOCX 28 kb) [file 12888_2017_1327_MOESM1_ESM.docx]

S1. Study Protocol

**Study Protocol:** Reducing secondary distress in violence researchers: a randomised trial of the effectiveness of group debriefings.

**Introduction:**

Vicarious trauma (VT) is defined as “the transformation of the therapist’s or helper’s inner experience as a result of empathetic engagement with survivor clients and their trauma material” [1]. Empathetic listening to a victim’s traumatic experiences may evoke ‘intense emotions such as profound sadness, helplessness, frustration, and anger’ (Woolhouse et al., 2012). This emotional distress can in turn lead to VT characterised by a disruption in a therapist’s or helper’s view of themselves, others and the world[1].

Vicarious trauma have been described in trauma councillors, social workers and other health professionals [2, 3]. It was also recently described in sexual violence researchers[4] and in qualitative researchers who conduct interview-based research with children who have suffered abuse [5]. Various studies indicate the effectiveness of group psychological debriefings and other interventions in alleviating the effects of vicarious distress in professionals [5, 6].

In the light of the known risk factors, this study will be investigate the prevalence of emotional distress as measured by a ‘state’ measure of symptoms of depression and anxiety, and vicarious trauma, in Ugandan interviewers. These interviewers are engaged in interview-based research with children who experienced abuse. The effect of group debriefings on mitigating the experience of vicarious trauma will also be assessed. This study will be nested within a larger on-going clustered randomised control trial commissioned to evaluate the impact of the ‘Good Schools Toolkit’ to prevent violence against children in Ugandan primary schools. The larger randomised control trial will employ 50 Ugandan interviewers over a period of 5 weeks to interview 3700 children at risk of abuse at their school or home.

**Hypotheses:**

- Ugandan interviewers experience vicarious trauma/emotional distress as a result of empathetic listening to children who disclose their abuse, even if the exposure is over a short term (5 weeks).
- The interviewers’ emotional distress/vicarious trauma can be mitigated through group debriefing sessions and related strategies.

**Aim:** Assessing the effectiveness of group debriefings in mitigating the experience of vicarious trauma/emotional distress in Ugandan interviewers.

**Objectives:**

- To assess the prevalence of vicarious trauma/emotional distress in Ugandan interviewers as a result of empathetic listening while interviewing children who disclose abuse.
- To estimate the levels of vicarious trauma/emotional distress in those Ugandan interviewers participating in group debriefing sessions compared to the control group.
- To make recommendations considering the likelihood of vicarious trauma in researchers who conduct short term, interview-based surveys with children who have suffered abuse.
- To make recommendations regarding the effectiveness of elementary group debriefing strategies to mitigate vicarious trauma when utilised by lay facilitators in resource constrained settings.

**Sample selection:** The proposed study will be nested within a larger on-going clustered randomised control trial commissioned to evaluate the impact of the ‘Good Schools Toolkit’ on children’s experience of violence. A group of 50 Ugandan interviewers employed to interview school children for the randomised control trial will be invited to participate in the proposed study. Half of the interviewers recruited into the study will be randomly selected to participate in weekly group debriefing sessions aimed at mitigating vicarious trauma.

**Randomisation procedure:**  In a group meeting, all participating interviewers will be invited to place a piece of paper with their name into an opaque paper bag. The group will be informed that the first, thirds, fifth and so on names drawn from the bag will be allocated to the intervention group, and the second, fourth, sixth and so on names will be allocated to the control group. A person nominated by the group will then draw numbers out of the bag and interviewers will be allocated accordingly.

**Treatment group 1 (intervention):** Group 1 will receive a weekly group debriefing based on simple, yet effective vicarious trauma interventions as confirmed by evidence-based literature. In session 1, there will be a group discussion focused on expression and discussion of current personal experiences with the study. The next session will connect current experiences with individual’s own histories and life experiences. The last session will focus on societal and community responses to the issues raised and focus on constructive ways to address them. Care will be taken in each session to ensure that individuals are not pressured to disclose their experiences and that they can disclose their experiences anonymously if they wish (for example, by writing them on a piece of paper rather than saying them out loud).

**Treatment group 2 (control):** During the same time slot group 2 will be allocated to a weekly leisure or relaxation activity. These activities will consist of film showings or other similar activities. Group 2 will be afforded similar group debriefing for vicarious trauma at the end of the 5 week period post completion of the end-line survey.

**Procedures:** Written consent will be obtained from the study participants. Surveys will be administered to the interviewers of the Good Schools Toolkit Study at the trial field station. Although the study is nested within the Good Schools Toolkit Study, the intervention for the interviewers will be facilitated by Heidi Grundlingh. The questionnaire will be programmed into mobile phones and will be self-completed by the study interviewers, enabling them to do so in private locations. They will be identified only by their interviewer number, and data will be stored confidentially.

The questionnaire will be programmed into mobile phones and will be self-completed by the study interviewers, enabling them to do so in private locations. Data storage and management procedures for the larger Good Schools Study will be followed. All data will be fully anonymised before analysis so it will not be possible to identify individuals.

**Data management:**  Data will be stored with the rest of the Good Schools Study data in accordance with the same procedures on a secure LSHTM server. As per Good Schools agreement, after 10 years anonymised data will be made publicly available.

**Safety:** All interviewers are given the contact information for separate services that they may contact at any time without the study team knowing. Some interviewers may need additional counselling if severe secondary trauma or vicarious trauma is elicited during the proposed study. Study participants will be referred for professional counselling in this case.

**Data collection tools:** A baseline and end-line survey will be administered to all recruited Ugandan interviewers. The surveys will assess interviewer characteristics and the two treatment groups’ experience of vicarious trauma/emotional distress. The outcome of interest will be assessed through the SRQ-20 form which measures levels of symptoms depression or anxiety (emotional distress measure). The Vicarious Trauma Scale (VTS) will be used during the end-line survey [3].

**Primary outcome:** SRQ-20 score. Changes in individual level SRQ-20 scores between baseline and endline, modelled as a continuous variable.

**Secondary outcomes:** Vicarious trauma scores. Levels of vicarious trauma at endline, modelled as a continuous variable.

**Statistical power:** We recognise that we will have limited statistical power to detect a difference between groups, hence this will be viewed as an exploratory study. We expect low attrition, based on the previous experience of hiring interviewers for the baseline Good Schools Study. Allowing for a loss of 4 interviewers (2 per arm), at a 5% level of significance, we will have approximately 90% power to detect a 1.25 point difference in mean base and end-line SRQ20 scores.

**Data analysis methods:** As the study will only recruit a relatively small sample of up to 50 participants, this will be an exploratory trial. Changes in SRQ-20 between baseline and endlines surveys for each interviewer will be computed, and the mean change in treatment group 1 and treatment group 2 will be compared using a t-test. Other outcomes will be compared across groups in a similar manner

References.

1. Pearlman, L.A. and K.W. Saakvitne, *Trauma and the therapist: Countertransference and vicarious traumatization in psychotherapy with incest survivors*. 1995: WW Norton & Co.

2. Cohen, K. and P. Collens, *The Impact of Trauma Work on Trauma Workers: A Metasynthesis on Vicarious Trauma and Vicarious Posttraumatic Growth.* Psychological Trauma-Theory Research Practice and Policy, 2013. **5**(6): p. 570-580.

3. Aparicio, E., L.M. Michalopoulos, and G.J. Unick, *An examination of the psychometric properties of the vicarious trauma scale in a sample of licensed social workers.* Health and Social Work, 2013. **38**(4): p. 199-206.

4. Coles, J., et al., *A qualitative exploration of researcher trauma and researchers' responses to investigating sexual violence.* Violence Against Women, 2014. **20**(1): p. 95-117.

5. Coles, J. and N. Mudaly, *Staying Safe: Strategies for Qualitative Child Abuse Researchers.* Child Abuse Review, 2010. **19**(1): p. 56-69.

6. Everly, G.S., S.H. Boyle, and J.M. Lating, *The effectiveness of psychological debriefing with vicarious trauma: A meta-analysis.* Stress Medicine, 1999. **15**(4): p. 229-233.
