# Supplementary material for: Secondary distress in violence researchers: a randomised trial of the effectiveness of group debriefings
Source: BMC Psychiatry. 2017 Jun 2;17:204. doi: 10.1186/s12888-017-1327-x (PMC5455179; doi:10.1186/s12888-017-1327-x)
Supplement: Supplementary file 3 — Outline: group debriefing sessions for secondary distress. (DOCX 13 kb) [file 12888_2017_1327_MOESM3_ESM.docx]

S2. Outline: group debriefing sessions for secondary distress.

**GROUP DEBRIEFING FOR SECONDARY DISTRESS**

**Outline Session 1 (Current personal reactions to trauma work)**

- Ice Breaker: Encouraging equal participation.
- Group Contract: Group agreement on voluntary participation, group confidentiality and mutual trust and respect.
- Phased, step-wise small and big group discussions to facilitate :
  - Story-telling of accounts of distressing primary trauma cases encountered by the researchers.
  - Researchers’ verbalisation of their emotional reaction to various distressing stories.
- Psycho-education: Symptoms of emotional distress or secondary trauma.
- Psycho-education: Recommended coping mechanisms as suggested by the group members and facilitator.

**Outline Session 2 (Personal history of negative discipline)**

- Role Play: Current personal reactions to trauma work and ‘my own history of negative discipline’.
- Phased, step-wise small and big group discussions to facilitate:
  - Brief story-telling of new accounts of distressing primary trauma cases encountered by the researchers in the past week.
  - Writing exercise and appropriate story-telling of personal history of negative discipline as a child. (As a means of group learning, researchers instructed to share only appropriate stories if they felt it can be illustrative to other group members AND they have come to terms with the experience.)
- Balloon demonstration and discussion: Resilience definition and practical application to life and work situations.
- Psycho-education: Additional recommended coping mechanisms as suggested by the group members and facilitator.

**Outline Session 3 (Personal agency to facilitate constructive societal and community responses to violence and** **trauma.)**

- Ice Breaker: “Trust Fall” activity to encourage trust among group members.
- Phased, step-wise small and big group discussions to facilitate:
  - Review of resilience definition and practical application to life and work situations.
  - Writing exercise and appropriate story-telling of personal history of developing resilience as a child or adult.
  - A discussion of how group members can help others in their immediate community to become resilient even though they have experienced violence. (Encouraging personal agency for an improved sense of control and well-being.)
- Summary of issues raised in the three sessions: Encouragement to identify personal adverse emotional responses, apply personal and recommended coping mechanisms to develop resilience and finally employing personal agency to support resilience in others.
